# Incorporating PSMA-Targeting Theranostics Into Personalized Prostate Cancer Treatment: a Multidisciplinary Perspective

**DOI:** 10.3389/fonc.2021.722277

**Published:** 2021-07-28

**Authors:** Thomas S. C. Ng, Xin Gao, Keyan Salari, Dimitar V. Zlatev, Pedram Heidari, Sophia C. Kamran

**Affiliations:** ^1^Division of Nuclear Medicine and Molecular Imaging, Department of Radiology, Massachusetts General Hospital, Harvard Medical School, Boston, MA, United States; ^2^Division of Hematology and Oncology, Department of Medicine, Massachusetts General Hospital, Harvard Medical School, Boston, MA, United States; ^3^Department of Urology, Massachusetts General Hospital, Harvard Medical School, Boston, MA, United States; ^4^Department of Radiation Oncology, Massachusetts General Hospital, Harvard Medical School, Boston, MA, United States

**Keywords:** PSMA, PET, prostate cancer, radiation, theranostics, therapy, molecular imaging

## Abstract

Recent developments in prostate-specific membrane antigen (PSMA) targeted diagnostic imaging and therapeutics (theranostics) promise to advance the management of primary, biochemically recurrent, and metastatic prostate cancer. In order to maximize the clinical impact of PSMA-targeted theranostics, a coordinated approach between the clinical stakeholders involved in prostate cancer management is required. Here, we present a vision for multidisciplinary use of PSMA theranostics from the viewpoints of nuclear radiology, medical oncology, urology, and radiation oncology. We review the currently available and forthcoming PSMA-based imaging and therapeutics and examine current and potential impacts on prostate cancer management from early localized disease to advanced treatment-refractory disease. Finally, we highlight the clinical and research opportunities related to PSMA-targeted theranostics and describe the importance of multidisciplinary collaboration in this space.

## Introduction

Prostate cancer is the second most common malignancy in men and the fifth leading cause of cancer-related death worldwide (1). Localized indolent disease has a good prognosis; however, advanced localized, recurrent and metastatic disease often portend poor outcomes (1, 2). Prostate-specific membrane antigen (PSMA) is increasingly appreciated as a promising imaging and therapeutic target for prostate cancer (3). As these agents become FDA-approved and clinically available, opportunities and challenges will arise to incorporate them appropriately into the management armamentarium for prostate cancer. Success in this endeavor will require coordination and collaboration among the clinical stakeholders in prostate cancer management, including imaging physicians, medical oncologists, urologists and radiation oncologists. In this review, we provide a multidisciplinary viewpoint of how PSMA-targeting agents will advance clinical management of prostate cancer. We outline the PSMA-targeted agents for imaging and therapy and their roles in the management of both localized and metastatic disease. Finally, we identify opportunities for cross-specialty collaboration to advance the utility of PSMA-targeted agents for prostate cancer management.

## PSMA: A Promising Imaging/Therapeutic Target

PSMA is expressed at 100–1000-fold higher levels in prostate cancer compared to healthy prostate tissue, and importantly, shows highest expression in high-grade and castration-resistant prostate cancer (3, 4). Multiple studies have demonstrated correlation between PSMA expression and prostate cancer aggressiveness (5), Gleason score (6), metastatic potential (7) and castration resistance (8, 9), suggesting that PSMA is a promising imaging/therapeutic target.

## PSMA-Targeted Imaging Agents

The first FDA-approved molecular imaging agent developed to target PSMA was the radiolabeled monoclonal antibody indium-111 (^111^In)-capromab pendetide (ProstaScint) for single-photon emission computed tomography (SPECT) imaging detection of sites of biochemical recurrence (10). Clinical adoption of ProstaScint has remained low due to the relatively poor resolution of SPECT imaging as well as limited sensitivity due to an unfavorable biodistribution and the antibody targeting an intracellular epitope of PSMA (11–13).

Several SPECT-imaging agents targeting PSMA were developed after ProstaScint, including agents labeled with ^99m^Tc (14–16) and ^123^I (17). However, more recent attention has been focused on positron emission tomography (PET) agents, which offer higher sensitivity and spatial resolution compared to SPECT (4, 18).

**Figure 1:**
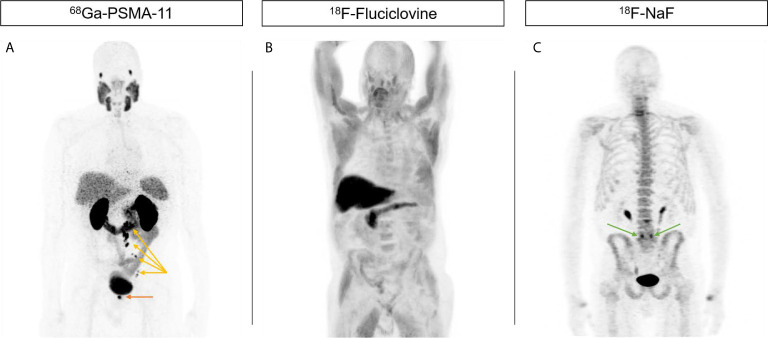
Increased sensitivity of PSMA-targeted imaging compared to current alternatives. 71-year-old male presented initially with T3 N0 M0 Gleason 4 + 5 = 9 PSA 9.42 prostate adenocarcinoma who declined local therapy and was managed with ADT alone, subsequently with castration-resistant progression. **(A)** ^68^Ga-PSMA-11 PET showing focal uptake in the right prostate bed (orange arrow) as well as left pelvic and retroperitoneal nodes (yellow arrows). **(B)** ^18^F-Fluciclovine PET do not show any abnormal uptake. **(C)** ^18^F-NaF PET do not show any abnormal uptake suspicious for metastases. Uptake at L5/S1 facets is due to degenerative change (green arrows).

The pharmacokinetics of small molecules with their fast clearance and good tumor penetration results in a high tumor to background ratio (19). These properties make them ideal as imaging agents. **Table 1** lists the most common PSMA-targeting small molecule agents that are actively used in trials and clinically worldwide. At the time of writing, two of these agents, ^68^Ga-PSMA-11 and ^18^F-DCFPyL, have been FDA-approved (but awaiting CMS approval). Beyond favorable imaging profiles (20), these agents have been demonstrated in multiple retrospective and prospective studies to be superior compared to standard cross-sectional imaging, (CT/MRI) (21, 22) nuclear medicine assays (bone scintigraphy) (23, 24), ^18^F-fluciclovine (25–29), ^11^C-choline PET/CT (30, 31), and other modalities (32, 33) for characterizing disease burden across the spectrum of the disease (**Figure 1**). Applications include the localized disease setting for both intraprostatic localization and staging (34–36), detection of lesions during biochemical recurrence (22, 37), and for stratification and treatment monitoring in metastatic disease (38). Furthermore, PSMA-targeted imaging has shown synergy with other modalities such as multiparametric prostate MRI (39, 40) and FDG-PET for improved characterization of disease burden (41) and image guidance for bone biopsies (42).

**Table 1:** Clinically relevant PSMA-targeted imaging and radio-therapeutic agents (Active at the time of review).

Agent name	Current use	Radioisotope	Target backbone	Notes	Clinical Trials for lesion detection	Therapy-based clinical trials
PSMA-11	Diagnostic	^68^Ga, ^18^F	Urea	FDA-approved in 2020, but unclear if there will be reimbursement currently. Kit-based formulation also available.	NCT04846894	NCT04279561 (Androgen receptor inhibitors)
					NCT04831541	NCT03977610 (ADT)
					NCT04462926	NCT04264208 (brachytherapy)
					NCT04216134	NCT03949517
					NCT04483414	(HIFU, HDR)
					NCT04147494	NCT04794777 (Salvage radiotherapy)
					NCT04279561	NCT04086966 (RT planning)
					NCT04179968	
					NCT03809078 (Surgical guidance)	
					NCT03396874	
					NCT03187990	
					NCT03429244	
					NCT04176497	
					NCT03756077	
					NCT03762759	
PSMA-617	Diagnostic and Therapy	^68^Ga, ^64^Cu, ^177^Lu, ^225^Ac, ^44^Sc	Urea	Improved binding affinity and internalization into cells compared to PSMA-11.	NCT04796467	NCT04597411 (225Ac)
					NCT03606837	NCT03805594
						(^177^Lu + pembro)
						NCT04430192
						NCT03780075
						NCT04343885
						NCT03874884
						NCT04663997
						NCT03454750
						NCT04419402
THP-PSMA	Diagnostic	^68^Ga	Urea	Kit-based formulation, but lower tumor uptake compared to PSMA-11	NCT04158817	
PSMA-I&T	Diagnostic and Therapy	^68^Ga, ^177^Lu	Urea	Similar performance characteristics as PSMA-11 and PSMA-617		NCT04188587
						NCT04297410
						NCT04443062
PSMA-I&S	Diagnostic	^99m^Tc	Urea	SPECT agent	NCT04832958	
					NCT04857502	
					NCT03857113	
^18^F -DCFBC	Diagnostic	^18^F	Urea	Poor blood pool clearance		
^18^F -DCFPyL	Diagnostic	^18^F	Urea	FDA-approved in 2021, but unclear if there will be reimbursement currently. Similar performance as PSMA-11. Disease detection rate of 59-66% and change in management of 63.9%.	NCT03739684	NCT04457245 (RT)
					NCT03800784	NCT04461509 (HIFU)
					NCT03793543	NCT03253744 (SBRT)
					NCT03824275	NCT03972657 (antiCD28)
					NCT04727736	NCT03525288 (RT)
					NCT04390880	
					NCT03232164	
					NCT03585114	
					NCT03160794	
					NCT03173924	
					NCT02899312	
					NCT02420977	
					NCT03594760	
					NCT03976843	
					NCT03392181	
					NCT04017104	
					NCT04700332	
					NCT04266392	
					NCT03718260	
					NCT03619655	
					NCT03860987	
					NCT04030338	
^18^F -PSMA-1007	Diagnostic	^18^F	Urea	Reduced renal and increased hepatobiliary excretion compared to other agents, but also increased benign tissue uptake	NCT04487847	
					NCT04239742	
					NCT03876912	
					NCT04794777	
FrhPSMA-7	Diagnostic and Therapy	^18^F, ^177^Lu	Urea	Radio hybrid. Low bladder retention, Disease detection rate of 71% at low PSA levels.	NCT04186819	
					NCT04186845	
CTT1057	Diagnostic	^18^F	Phosphoramidate	Irreversible binding to PSMA, lower radiation dose to kidneys and salivary glands compared to urea agents. Potentially higher tumor-to-background ratio.	NCT03822871	
J591	Therapy	^225^Ac, ^177^Lu	Monoclonal antibody			NCT04576871
						NCT04506567
						NCT00859781
Rosopatamab	Therapy	^177^Lu	Monoclonal antibody			NCT04876651

ADT, androgen deprivation therapy; HDR, high dose radiation; HIFU, high intensity frequency ultrasound; RT, Radiation therapy; SBRT, stereotactic body radiation therapy.

## Role of PSMA Imaging in Localized Disease

Accurate staging is critical for risk stratification and treatment decisions. Surgery and radiation therapy are curative treatments for localized disease, offer potential cure for biochemically recurrent disease (i.e., salvage radiotherapy or salvage prostatectomy), and can offer durable control in the oligometastatic disease setting. To the extent PSMA imaging can identify micrometastatic disease and reclassify clinical stage, patient selection for local therapies can be expected to improve. Further, the success of radiation largely centers on accurate identification and encompassing of disease within a radiation field in the setting of localized or salvage radiation, or to precisely target disease with stereotactic ablative radiation therapy (SABR) for patients with oligometastatic prostate cancer. Conventional imaging has low sensitivity and low specificity for detection of prostate cancer spread. Thus, PSMA imaging is being explored to determine its role in early stage disease, including for accurate assessment of intraprostatic tumor burden, with higher PSMA uptake previously shown to be associated with histological identification of focal lesions (39, 43, 44). This can guide focal SABR escalation at these sites (45, 46).

### High-Risk Disease

Early data exploring the role of PSMA PET/CT in high-risk disease suggest that it can lead to changes in treatment decisions. The proPSMA trial recruited men with high-risk localized prostate cancer randomized to either conventional imaging or PSMA PET/CT as first-line imaging, followed by second-line cross-over imaging for patients with fewer than three distant metastases (21). PSMA PET/CT as first-line imaging led to change in management in 28% of patients (compared to 15% following conventional imaging), half of which comprised a change in surgical or radiotherapy technique. In patients who underwent second-line imaging, PSMA PET/CT similarly led to a change in management in 25% of patients, compared to only 5% following conventional imaging. A separate retrospective study of 138 prostate cancer patients who underwent ^68^Ga-PSMA-PET/CT imaging at initial diagnosis evaluated the number and anatomical location of PSMA-positive lymph nodes (47). Overall, 441 PSMA-positive lymph node metastases were identified (most frequently of which were internal iliac lymph nodes [25%]). The PSMA-positive lymph nodes were mapped onto a CT planning scan and the standard pelvic radiotherapy fields were overlaid on top for comparison. Extending the cranial border of the pelvic field from L5/S1 to L4/L5 increased accuracy of covering potentially involved nodes. Another recent study used data from two prospective trials with PSMA PET/CT imaging in high-risk individuals with cN0M0 disease per conventional imaging to develop a nomogram to help identify high-risk patients who might benefit from the addition of a PSMA PET/CT (48).

### Localized Salvage Therapy

Biochemical recurrence after radical prostatectomy or primary radiation can potentially be cured with localized salvage therapy such as pelvic-targeted radiation or salvage prostatectomy (49). Biochemical recurrence can now be detected at earlier PSA values, with the definition of failure at 0.2 ng/mL (50). At these low PSA levels, conventional imaging has poor sensitivity for detecting sites of recurrence. PSMA imaging has been shown to be more sensitive in this setting in multiple prospective studies (27, 37, 51–53). The enhanced detection of local and distant lesions with PSMA-targeted imaging has ramifications for treatment planning, including choice of localized *vs*. systemic therapy. For example, a study in 79 radio-recurrent patients using ^18^F-DCFPyL PET/CT not only showed superior disease detection compared to conventional imaging (87% *vs*. 67% overall, 30% *vs*. 15% for identifying distant metastases), but changed the proposed management in 43% of patients (54). However, it currently remains unknown whether these changes in management are appropriate or will improve overall disease outcomes.

### Oligometastatic Disease

The role of surgery and radiation therapy is evolving in the management of low-burden metastatic disease, also known as oligometastatic disease. Early data suggest that aggressive radiation targeted at metastatic lesions may improve outcomes (55–57). PSMA imaging may contribute to increased detection of metastatic disease and thus increased number of patients classified as oligometastatic prostate cancer. A phase II trial evaluating use of metastasis-directed therapy (MDT) to PSMA-defined oligorecurrent prostate cancer demonstrated that, of 37 patients undergoing MDT (stereotactic ablative body radiotherapy [SABR] or surgery), 22% were rendered biochemically disease-free (58). The ORIOLE trial, a randomized phase 2 trial, evaluated men with 1-3 lesions defined on conventional imaging, randomizing to standard-of-care treatment *versus* SABR to all detectable lesions. Patients who underwent SABR also had PSMA PET/CT at baseline and day 180. Overall, 16/36 SABR patients had 1 or more PSMA-positive lesions that were not included as part of the SABR-directed therapy. Of those who had no untreated lesions, the proportion with progression at 6 months was 1/19 (5%) compared to 6/16 (38%) with any untreated lesion. Men who had all PSMA-positive lesions treated were less likely to have new lesions at 6 months (3 of 19, 15.8% *versus* 10 of 16, 62.5%, p=0.006) (59). Taken together, these data support that aggressive metastasis-directed treatment to all PSMA PET-avid lesions may be curative in a subset of patients with low-burden metastatic disease.

### Surgical Guidance With PSMA-Imaging

Molecular imaging approaches are increasingly being adopted for surgical guidance (60). Pelvic lymph node dissection (PLND) is the standard approach for nodal staging or management of local lymphatic metastases (61). PSMA-targeted radiolabeled and fluorescent probes are being tested for identifying lymph node metastases intraoperatively during PLND, for confirming appropriate surgical margins, and for correlation with pathological assessment (62–65). These approaches may improve surgical outcomes by increasing the likelihood that all clinically significant disease is resected at the time of surgery.

## Therapeutic Role of PSMA in Metastatic Disease

### PSMA-Targeted Radioligand Therapy

Systemically delivered radiotherapies already play a key role in metastatic prostate cancer management, especially with the use of ^223^Radium for management of osseous lesions (66). PSMA-targeted radiotherapies are poised to offer an even more impactful alternative, being effective for both PSMA-expressing bone and soft tissue metastases (67). To date, the most tested PSMA-targeted agent is ^177^Lu-PSMA-617, among other agents outlined in **Table 1**, with several clinical studies showing significant treatment response, both by imaging and PSA monitoring (68–70). The largest randomized phase III trial comparing ^177^Lu PSMA-617 to standard of care alone in 831 patients with advanced metastatic castration-resistant prostate cancer (mCRPC) (VISION) demonstrated that ^177^Lu PSMA-617 significantly improves overall survival (OS, median, 15.3 *vs*. 11.3 months) and progression-free survival (rPFS, median, 8.7 *vs*. 3.4 months) in patients with PSMA-positive mCRPC (71, 72). Based on the promising results of this trial, regulatory approval for this agent is expected to be imminent. Another randomized phase II trial (TheraP) demonstrated that ^177^Lu PSMA-617 compared with cabazitaxel in men with mCRPC led to a higher PSA response and fewer grade 3 or 4 adverse effects (73). Several studies have also extended the use of these agents for management of micrometastases in the setting of localized disease (74) and oligometastases (75). Correlation with PSMA imaging is key for patient stratification since patients with high PSMA expression level and low tumor heterogeneity show better outcomes (76, 77).

PSMA radioligand therapy is an area of active investigation, with most notable areas focused on testing various choices of radionuclides and ligands to improve outcomes and reduce toxicity (78). For instance, several radiopharmaceuticals have been engineered to enable radiolabeling of the same ligand using both imaging and therapeutic radionuclides, allowing an accurate pharmacokinetic readout using imaging prior to radioligand therapy (75) (4). Other areas of investigation focus on the development of therapeutic agents with more favorable pharmacokinetics for therapeutic payload delivery such as antibody constructs with longer biological half-lives and different organ toxicity profiles (79, 80). Additionally, the optimal choice of radionuclides is also being assessed. Commonly used radionuclides including ^177^Lu and ^90^Y for PSMA-therapy predominantly exert their cytotoxic actions *via* beta particle emission, with spatial range of action on the order of mm. Alpha particles, such a ^225^Ac or ^209^Pb, can confer higher linear energy transfer (up to 20x) compared to beta particles, but act on a shorter spatial range (81). ^225^Ac-PSMA-617 alone or in tandem with ^177^Lu-PSMA-617 have been studied clinically, with promising results (82). Auger emitters, which impart high energy at a shorter range than alpha particles, may also be useful in the setting of micro-metastases (83). Further preclinical and clinical studies are needed to understand and optimize the interplay between these design parameters, and their effects on efficacy.

### PSMA-Targeted Bispecific Agents

Multiple PSMA-targeted bispecific molecules have advanced to early phase clinical evaluation in patients with mCRPC. These antibody-derived bispecific molecules bind to PSMA and a T-cell-specific antigen such as CD3 or CD28, resulting in activation of T-cell response to PSMA-expressing prostate cancer cells. PSMA-targeted bispecific agents are being developed as monotherapies and in combination with immune checkpoint inhibitors.

Pasotuxizumab (AMG 212) is a bispecific T-cell engager (BiTE) engineered to engage PSMA and CD3 and demonstrated reasonable tolerability, immunogenicity, and clinical activity in a phase 1 dose-escalation study in mCRPC patients (84). PSA declines ≥50% (PSA_50_) occurred in 29% and 19% of patients treated with subcutaneous and intravenous dosing, respectively, including 2 long-term responders (11-17 months to tumor progression). Pasotuxizumab was limited by a short half-life, and a half-life extended anti-PSMA x CD3 BiTE acapatamab (AMG 160) was developed for further clinical evaluation. Preliminary results from the phase 1 study of acapatamab in heavily pretreated mCRPC patients showed promising activity and manageable toxicity (85). PSA_50_ responses were seen in 34% of evaluable patients, including a patient who previously progressed on lutetium-PSMA therapy. Cytokine release syndrome (CRS) was observed in 91% of patients, but most cases were grade 1-2 and decreased in severity after cycle 1. Combination therapy with anti-PD-1 immune checkpoint inhibitors, abiraterone, or enzalutamide is planned (85, 86).

HPN424 is a PSMA-targeting T-cell engager with three binding domains: anti-PSMA, anti-CD3, and anti-albumin for half-life extension (87). Preliminary results from the phase 1/2 study of HPN424 in mCRPC patients demonstrated PSA_50_ responses in 3 (5%) patients. In the highest fixed dose cohort evaluated to date, 3 of 7 patients had PSA declines and 1 patient had a confirmed partial response by RECIST. CRS events occurred in 63% of patients, with 4% of patients experiencing grade 3 CRS. The study continues in dose escalation.

Additional PSMA-targeted bispecific agents are entering the clinical setting. REGN5678 is a first-in-class human IgG4-based bispecific engineered to target PSMA and the T-cell costimulatory receptor CD28, and will be evaluated in a phase 1/2 first-in-human study as monotherapy and in combination with the anti-PD-1 antibody cemiplimab (88). TNB-585 and CCW702 are anti-PSMA x CD3 bispecific agents entering phase 1 evaluation (89, 90).

### PSMA-Targeted CAR-T

Chimeric antigen receptor (CAR) T cell therapies are a powerful class of genetically-engineered T cells with synthetic receptors that redirect their specificity, function, and metabolism, and represent a major advancement in the treatment of certain refractory hematologic malignancies (91). Prostate cancer serves as an attractive target for evaluation of CAR-T therapy in solid tumors due to the relative specificity of PSMA as target antigen. An early generation PSMA-targeted CAR-T was evaluated in a small phase 1 study that reported clinical partial responses in 2 of 5 mCRPC patients, with PSA declines of 50% and 70% (92). A second generation PSMA-targeted CAR-T demonstrated evidence of cytokine activation and prolonged stable disease for >6 months in 2 of 7 patients dosed (93).

More modern CAR-T therapies are now entering clinical evaluation for mCRPC patients, with some reporting very preliminary results to date. CART-PSMA-TGFβRDN cells involving autologous T cells engineered to express a dominant negative form of TGFβRII and a CAR with specificity to PSMA reported PSA_50_ decreases in 2 of 3 patients with one-month follow-up, including a patient with >95% PSA decline (94). However, one patient developed grade 2 CRS that progressed to fatal encephalopathy and multi-organ failure despite aggressive immunosuppressive therapy. A second CART-PSMA-TGFβRdn study has reported early results with PSA_50_ decline in 1 of 10 patients (98% decline) and PSA_30_ decline in 3 additional patients (95). Grade 2+ CRS was seen in 5 of 7 patients treated at higher dose. However, the therapy was associated with lethal neurotoxicity and sepsis. P-PSMA-101 is an autologous CAR-T product being evaluated in the U.S. (NCT04249947), while several PSMA-targeted CAR-T products are in clinical trials in China (NCT04053062, NCT04768608, NCT04429451). PSMA-imaging has also been harnessed as a means to track CAR-T trafficking (96).

### PSMA-Targeted Antibody-Drug Conjugates

Antibody-drug conjugates (ADCs) comprise a monoclonal antibody binding to a target antigen that is highly specific to tumor cells, a synthetic linker domain, and a potent cytotoxic chemotherapy payload (97). ADCs can deliver chemotherapeutics in a more targeted manner to tumor cells, while sparing normal cells. PSMA represents a rationale target for the development of ADCs.

MLN2704, PSMA ADC, and MEDI3726 are three PSMA-targeted ADCs that have undergone clinical investigation to date. MLN2704 is comprised of a de-immunized anti-PSMA monoclonal antibody (J591) with high affinity to the external domain of PSMA complexed *via* a thiopentanoate linker to maytansinoid-1, a potent anti-microtubule chemotherapeutic (98). PSMA ADC is a fully human immunoglobulin G1 anti-PSMA monoclonal antibody complexed to the anti-mitotic agent monomethyl auristatin E *via* a valine-citrulline linker, which is more stable than thiol linkers in plasma (99). MEDI3726 is comprised of J591 conjugated to the DNA cross-linking agent pyrrolobenzodiazepine (100). These PSMA-targeted ADCs have been evaluated in separate early phase clinical trials in mCRPC patients, with MLN2704 and PSMA ADC treatments associated with PSA_50_ response in 8% and 14% of patients, respectively, while MEDI3726 reported a modest 12% composite response rate involving radiographic, PSA_50_, and circulating tumor cell (CTC) responses. However, these PSMA-targeted ADCs have been limited by neuropathy, skin toxicities, and cytopenias. Nonetheless, the clinical studies further validate PSMA as a therapeutic target in mCRPC, and future development of ADCs may focus on improving synthetic linkers that limit deconjugation of the chemotherapeutic payload outside of the tumor microenvironment.

## Multidisciplinary Opportunities and Challenges in the Era of PSMA-Targeted Prostate Cancer Management

PSMA-targeted imaging and therapy are poised to play key roles in the management of prostate cancer. Evaluation of their clinical utility will require high-level evidence from prospective clinical studies (101). Precision medicine principles guided by theranostics should be incorporated in the design of these trials, and will require collaboration across radiology/nuclear medicine, urology, medical and radiation oncology. Standardized acquisition methods, and interpretation criteria of PSMA-based imaging exams, such as with recently proposed criteria like the PROMISE staging system (102) or PSMA-RADS (103) will be paramount in this regard. In addition, collaborative efforts at both pre-clinical and clinical levels to examine combination treatments involving the different PSMA-targeting modalities are vital to understanding their optimal role in the treatment armamentarium for prostate cancer.

In summary, exciting opportunities abound with the multiple PSMA-targeted imaging and therapy agents in the clinical pipeline. Collaboration across the different clinical disciplines in the prostate cancer management team will be crucial to maximize the potential of these agents.

## Author Contributions

All authors contributed to the article and approved the submitted version.

## Funding

This work is supported in part by a Thrall Innovation Grant from the Department of Radiology, Massachusetts General Hospital (TN) and 1K08CA249047-01(PH).

## Conflict of Interest

The authors declare that the research was conducted in the absence of any commercial or financial relationships that could be construed as a potential conflict of interest.

## Publisher’s Note

All claims expressed in this article are solely those of the authors and do not necessarily represent those of their affiliated organizations, or those of the publisher, the editors and the reviewers. Any product that may be evaluated in this article, or claim that may be made by its manufacturer, is not guaranteed or endorsed by the publisher.
